# Association Between Vasomotor Symptoms and Ischemic Stroke: A Case‐Control Study

**DOI:** 10.1002/brb3.71104

**Published:** 2025-11-26

**Authors:** Xiaoqing Jiang, Qi Qiu, Yue Wang, Yan Xie, Ling Yuan, Yuanmei Zhao, Chunyu He

**Affiliations:** ^1^ Chengdu Medical College Chengdu China; ^2^ The First Affiliated Hospital of Chengdu Medical College Chengdu China

**Keywords:** hot flashes, ischemic stroke, menopause, vasomotor symptoms

## Abstract

**Background:**

Ischemic stroke (IS) represents the most prevalent subtype of cerebrovascular disease. Although vasomotor symptoms (VMS) have been linked to cardiovascular disease (CVD), their association with specific stroke subtypes remains unclear.

**Methods:**

This multicenter case‐control study included 358 postmenopausal women, consisting of 179 patients with IS and 179 age‐matched controls. Participants were assessed for VMS frequency and severity, lifestyle behaviors, and established vascular risk factors. Stroke severity was measured using the National Institutes of Health Stroke Scale (NIHSS).

**Results:**

Patients with IS exhibited a higher prevalence of VMS during the perimenopause (*p* < 0.001) and in the past week (*p* = 0.001) compared to controls. IS patients also reported more frequent VMS during both the perimenopausal period (*p* = 0.001) and the past week (*p* = 0.014). After multivariable adjustments, both perimenopausal VMS (OR: 2.16, 95% CI: 1.42–3.30, *p*  <  0.001) and the past week VMS (OR: 2.06, 95% CI: 1.33–3.26, *p* = 0.001) remained independently associated with IS. Correlation analysis within the patient group showed no correlation between NIHSS scores and either the modified Kupperman index or the hot flush rating scale scores (*p* > 0.05). The area under the ROC curve for perimenopausal and recent hot flashes was 0.809 (*p* < 0.001), indicating substantial predictive value.

**Conclusions:**

VMS independently predicts the risk of IS. Women who experience frequent VMS should undergo targeted surveillance and early preventive strategies.

## Graphical abstract

Menopause is a risk factor for cardiovascular disease in women, with a significant increase in stroke incidence following menopause. Vasomotor symptoms are characteristic manifestations of the perimenopausal and postmenopausal phases. This case‐control study demonstrates that menopausal vasomotor symptoms are independently associated with an increased risk of ischemic stroke and may serve as predictive markers for ischemic stroke in women.

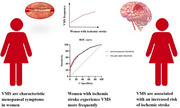

## 1 Introduction

Ischemic stroke (IS) is a major contributor to global mortality and disability, its incidence increasing with age, particularly among women aged 50 to 69 years (Ding et al. 2022). This trend may be attributable to sex‐specific reproductive factors (Bushnell et al. 2014). Previous studies have identified menopause as a risk factor for cardiovascular disease (CVD) in women, with a marked increase in stroke incidence following menopause (Welten et al. 2021). Additionally, factors such as menopausal hormone therapy (Mikkola et al. 2015), age at menopause (Zhu et al. 2019), and the method of menopause (Poorthuis et al. 2022) have also been linked to an increased risk of stroke. However, the relationship between menopausal symptoms and stroke remains insufficiently understood.

Hot flashes and night sweats, collectively known as vasomotor symptoms (VMS), are hallmark symptoms of the menopausal transition and postmenopause. Approximately 50.3%–82.1% of women worldwide experience VMS (Dibonaventura et al. 2013). The prevalence of VMS increases in the 2 years preceding menopause, reaching its peak within the first year after menopause (Gold et al. 2006). Frequent or severe VMS typically persist for 7 to 10 years, whereas milder symptoms may last for a longer duration (Avis et al. 2015). VMS not only affect quality of life and psychological well‐being during early menopause (Choi et al. 2024; Nappi et al. 2023) but also have long‐term implications for cardiovascular health.

The relationship between VMS and CVD may be partly attributed to VMS acting as an independent risk factor (Muka et al. 2016), and partly to adverse changes in established cardiovascular risk factors, such as hypertension (Kagitani et al. 2014) and diabetes (Gray et al. 2018), which are also linked to VMS. Furthermore, VMS are associated with markers of subclinical CVD, including endothelial dysfunction, increased carotid intima‐media thickness, and aortic calcification (Carson and Thurston 2023), which serve as early indicators of cardiovascular risk.

Current research on the relationship between VMS and stroke has three key limitations. First, existing studies employ cardiovascular events as the primary endpoint (Thurston 2024), without conducting separate analyses for stroke or differentiating between stroke subtypes. This approach may underestimate the effect of VMS on stroke risk and overlook potential differences based on stroke subtype. Second, the prevalence and characteristics of VMS exhibit significant geographic variation (Tang et al. 2020; Todorova et al. 2023), and studies specifically examining the link between VMS and stroke in Chinese populations are notably scarce. Third, the relationship between VMS and stroke risk requires further elucidation, particularly regarding the timing and severity of VMS symptoms (Bushnell et al. 2024). Therefore, our study aims to examine the association between the frequency of VMS across different menopausal stages and IS risk, adjusting for established stroke risk factors. Additionally, we aim to investigate whether the bothersomeness of VMS is associated with IS severity.

## 2 Methods

### 2.1 Study Population

Before inclusion in the study, all participants signed informed consent under the principles of the Declaration of Helsinki. The Medical Ethics Committee of Chengdu Medical College approved this study with code number “2024No.65.” Between October 2024 and June 2025, 179 stroke patients and 179 age‐matched non‐stroke controls were recruited from three hospitals in Chengdu, Sichuan Province: Chengdu Medical College First Affiliated Hospital, Chengdu Medical College Second Affiliated Hospital, and Chengdu Medical College Affiliated Hospital of Traditional Chinese Medicine.

**FIGURE 1:**
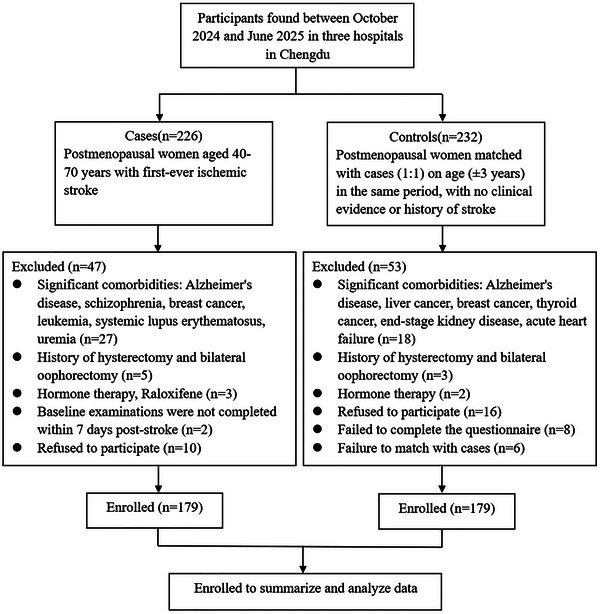
Flowchart of the study population selection process.

The majority of women experience menopause between the ages of 40 and 60 years (Du et al. 2020), with VMS potentially persisting for 10 years or longer. Therefore, inclusion criteria for the case group were as follows: postmenopausal women (defined as ≥ 12 months of amenorrhea without other pathological or physiological causes), aged 40 to 70 years, with a first‐ever confirmed IS. Exclusion criteria for the case group included: (1) significant comorbidities, including other intracranial organic diseases, severe psychiatric disorders, dementia or notable cognitive impairment, severe chronic hepatic or renal dysfunction, hormone‐sensitive malignancies, or systemic autoimmune diseases; (2) a history of hysterectomy and/or bilateral oophorectomy or other iatrogenic menopause; (3) current use of medications influencing VMS, including hormone therapy, selective estrogen receptor modulators, aromatase inhibitors, selective serotonin reuptake inhibitors, or serotonin‐norepinephrine reuptake inhibitors (Thurston et al. 2023); (4) inability to complete baseline examinations within the first week following stroke onset. Inclusion criteria for the control group were as follows: postmenopausal women matched to the case group by age (± 3 years), with no clinical evidence or history of stroke, as confirmed by MRI/CT. The exclusion criteria for the control group were identical to those for the case group, as shown in Figure 1.

### 2.2 Participant Clinical Information

Clinical data were collected through structured interviews and by reviewing the Electronic Medical Record systems from the three participating hospitals. Data collected included: age, time since menopause, education level (categorized as primary school or lower, junior or senior high school, college, or above), annual household income (categorized as <30,000, 30,000–50,000, >50,000 RMB), hypertension (previous physician diagnosis, use of antihypertensive medication, or an average of two blood pressure readings ≥140/90 mmHg at admission), diabetes (previous physician diagnosis or use of antidiabetic medication), dyslipidemia (previous physician diagnosis of hypercholesterolemia or hyperlipidemia, or use of lipid‐lowering medication), CVD history (any history of coronary heart disease, valvular heart disease, or atrial fibrillation), carotid stenosis (previous physician diagnosis or suggested by carotid CT or carotid ultrasound examination upon admission), current smoking (smoking ≥ 1 cigarette per day on average in the past 6 months), overweight/obesity (body mass index [BMI] ≥ 24 kg/m^2^, calculated from height and weight measured at admission), physical inactivity (engaging in moderate‐to‐vigorous physical activity less than 3 times per week for less than 30 min per session), current alcohol consumption (consuming ≥ 50 mL of alcohol per occasion at least once weekly for more than 6 consecutive months), and family history of stroke (stroke occurring in a first‐degree relative).

### 2.3 Diagnosis and Assessment of Stroke

All participants underwent standardized brain MRI or CT scans upon hospital admission to confirm the diagnosis of IS. In the case group, stroke severity was assessed using the National Institutes of Health Stroke Scale (NIHSS) score upon admission.

### 2.4 Assessment of VMS and Other Menopausal Symptoms

The modified Kupperman index (KI) (Ju et al. 2023) was used to retrospectively assess the occurrence and severity of VMS and other menopausal symptoms during the perimenopausal period (defined as the time from the onset of menstrual irregularities to 12 months after the final menstrual period). The modified KI is an internationally recognized tool for assessing menopausal symptoms, comprising 13 symptom items. Specifically, the hot flashes and night sweats items are scored based on reported frequency, categorized as: none, <3 times/day, 3–9 times/day, or ≥10 times/day. The total score (range: 0–63) is calculated by summing the products of each item's base score and severity rating. Higher total scores reflect greater overall severity of menopausal symptoms.

For participants reporting hot flashes during the perimenopausal period, the frequency and bothersomeness of hot flashes and night sweats in the week before hospital admission were assessed using the hot flush rating scale (HFRS) (Hunter et al. 2019). Items related to hot flash frequency and severity in the HFRS are rated on a 0–10 point scale, with 0 indicating “not a problem” and 10 indicating an “extremely severe problem.”

### 2.5 Statistical Methods

#### 2.5.1 Sample Size Calculation

Considering VMS as the primary exposure factor, the required sample size was calculated by PASS software. The calculation was based on a VMS prevalence of 39.7%, derived from a cross‐sectional study conducted in southeastern China (Lan et al. 2017), and an assumed odds ratios (OR) of 2.09, obtained from a pooled analysis of prospective cohort studies (Zhu et al. 2020), with α and β values set at 0.05 and 0.1. The minimum required sample size for the case group was calculated to be 154 participants. Ultimately, 179 participants were enrolled in both the case and control groups.

#### 2.5.2 Statistical Analysis

Statistical analyses were performed with SAS software (version 9.4 for Windows, SAS Institute, Inc., Cary, NC, USA). No data points were missing because both patients and controls were personally interviewed. Categorical variables are presented as frequencies and percentages, while continuous variables are presented as means and standard deviations (SD) or medians with interquartile ranges (IQR). The chi‐square test, independent two‐tailed *t*‐test, and Mann–Whitney U test were used for comparing cases and controls. Relationships between modified KI, HFRS, and IS severity were assessed using Pearson and partial correlation analyses. Binary logistic regression analyses were conducted to address potential confounding factors. The discriminative ability of VMS in predicting IS was evaluated with ROC curve analysis. The *p* value < 0.05 was considered statistically significant.

## 3 Results

### 3.1 Participant Baseline Characteristics

The baseline demographic and stroke risk factor characteristics of the 358 participants are summarized in Table 1. Participants in the IS group had a mean age of 60.53 ± 6.40 years and a mean duration of menopause of 9.80 ± 5.78 years. The corresponding values for the control group were 60.45 ± 6.11 years and 9.17 ± 5.51 years, respectively. Significant differences were observed between the two groups in age at menopause (*p* = 0.043), alcohol consumption (*p* = 0.010), hypertension (*p* < 0.001), diabetes mellitus (*p* < 0.001), CVD (*p* = 0.003), dyslipidemia (*p* = 0.032), carotid artery stenosis (*p* < 0.001), and family history of stroke (*p* = 0.001). Overall, participants exhibited low educational attainment, and the prevalence of current smoking was low.

**TABLE 1:** Demographic and stroke risk factor characteristics of the case and control groups.

	Controls (*N* = 179)	Ischemic stroke (*N* = 179)	*p* value
Age (M, SD)	60.45 ± 6.11	60.53 ± 6.40	0.906
Age at menopause (M; P25, P75)	52 (50–53)	51 (50–52)	0.043
Duration of menopause (M, SD)	9.17 ± 5.51	9.80 ± 5.78	0.286
Education level (*n*, %)			0.085
primary school or lower	83 (46.4)	104 (58.1)	
junior or senior high school	87 (48.6)	68 (38.0)	
college or above	9 (5.0)	7 (3.9)	
Annual income (RMB), (*n*, %)			0.372
<30,000	82 (45.8)	95 (53.1)	
30,000–50,000	61 (34.1)	51 (28.5)	
>50,000	36 (20.1)	33 (18.4)	
Current smoking (*n*, %)	5 (2.8)	6 (3.4)	0.759
Current alcohol consumption (*n*, %)	14 (7.8)	30 (16.8)	0.010
Hypertension (*n*, %)	51 (28.5)	108 (60.3)	<0.001
Diabetes (*n*, %)	15 (8.4)	57 (31.8)	<0.001
CVD (*n*, %)	10 (5.6)	27 (15.1)	0.003
Dyslipidemia (*n*, %)	49 (27.4)	68 (38.0)	0.032
Carotid stenosis (*n*, %)	18 (10.1)	58 (32.4)	<0.001
Overweight/Obesity (*n*, %)	72 (40.2)	88 (49.2)	0.089
Physical inactivity (*n*, %)	33 (18.4)	39 (21.8)	0.429
Family history of stroke (*n*, %)	29 (16.2)	55 (30.7)	0.001

Abbreviation: CVD, cardiovascular disease.

*p* value < 0.05 was considered statistically significant.

### 3.2 Menopausal Symptom Characteristics

Compared to controls, patients with IS had a higher prevalence of VMS during the perimenopausal period (*p* < 0.001) and in the past week (*p* = 0.001) (Table 2). Moreover, VMS episodes occurred more frequently among IS patients during both the perimenopausal period (*p* = 0.001) and the past week (*p* = 0.014). However, no significant difference was observed in the modified KI scores between the two groups (*p* > 0.05). Among participants who reported VMS, more than 80% experienced VMS either at the onset of menstrual irregularity or within the first year after menopause, and 33.2% reported symptom duration exceeding 10 years.

**TABLE 2:** Menopausal symptom characteristics in the case and control groups.

	Controls (*N* = 179)	Ischemic stroke (*N* = 179)	*p* value
KI (M, P25, P75)	10 (6, 14)	10 (6, 19)	0.112
VMS during the perimenopausal period (*n*, %)	76 (42.5)	110 (61.5)	< 0.001
VMS in the past week (*n*, %)	46 (25.7)	75 (41.9)	0.001
VMS frequency during the perimenopausal period (*n* %)			
None	103 (57.5)	69 (38.5)	0.001
<3 times/day	51 (28.5)	56 (31.3)	
3–9 times/day	19 (10.6)	43 (24.0)	
≥10 times/day	6 (3.4)	11 (6.1)	
VMS frequency in the past week (*n*, %)			0.014
None	133 (74.3)	104 (58.1)	
<3 times/day	28 (15.6)	45 (25.1)	
3–9 times/day	10 (5.6)	15 (8.4)	
≥10 times/day	8 (4.5)	15 (8.4)	
Onset time of VMS (*n*, %)			0.600
Regular menstruation	6 (7.9)	8 (7.3)	
Menstrual irregularity	43 (56.6)	53 (48.2)	
Less than 1 year after menopause	22 (28.9)	37 (33.6)	
More than 1 year after menopause	5 (6.6)	12 (10.9)	
Duration of VMS (*n*, %)			0.461
<5 years	50 (65.8)	65 (59.1)	
5–10 years	16 (21.1)	23 (20.9)	
>10 years	10 (13.2)	22 (20.0)	

**Abbreviations**: KI, modified Kupperman index; VMS, vasomotor symptoms.

*p* value< 0.05 was considered statistically significant.

### 3.3 Association Between VMS and IS Risk

In the unadjusted model, VMS during perimenopause (OR: 2.16, 95% CI: 1.42–3.30, *p* < 0.001) and the past week (OR: 2.06, 95% CI: 1.33–3.26, *p* = 0.001) were significantly linked to IS. These associations remained significant after adjustment for demographic factors and vascular risk factors. Higher VMS frequency during perimenopause was associated with increased IS risk, with frequencies of 3–9 times/day (OR: 2.88, 95% CI: 1.33–6.26, *p* = 0.008) and ≥ 10 times/day (OR: 3.58, 95% CI: 1.01–12.74, *p* = 0.049), showing the strongest associations. Similar results were observed for VMS frequency in the past week. Detailed OR and 95% CI are shown in Table 3.

**TABLE 3:** Binary logistic regression analysis of VMS and IS.

	Model 1		Model 2		Model 3	
	OR (95% CI)	*p* value	OR (95% CI)	*p* value	OR (95% CI)	*p* value
VMS during the perimenopausal period	2.16 (1.42–3.30)	< 0.001	2.21 (1.40–3.49)	0.001	1.93 (1.10–3.38)	0.022
VMS in the past week	2.06 (1.33–3.26)	0.001	2.66 (1.53–4.60)	< 0.001	2.47 (1.27–4.77)	0.007
VMS frequency during the perimenopausal period						
<3 times/day	1.64 (1.01–2.67)	0.047	1.62 (0.97–2.71)	0.068	1.42 (0.75–2.68)	0.278
3–9 times/day	3.38 (1.82–6.28)	< 0.001	3.64 (1.89–6.99)	< 0.001	2.88 (1.33–6.26)	0.008
≥10 times/day	2.74 (0.97–7.75)	0.058	3.41 (1.14–10.20)	0.028	3.58 (1.01–12.74)	0.049
VMS frequency in the past week						
<3 times/day	2.06 (1.20–3.52)	0.009	2.47 (1.34–4.57)	0.004	2.07 (0.99–4.31)	0.053
3–9 times/day	1.92 (0.83–4.45)	0.129	2.81 (1.09–7.24)	0.032	3.09 (1.05–9.04)	0.040
≥10 times/day	2.40 (0.98–5.87)	0.056	3.24 (1.23–8.55)	0.018	3.57 (1.16–11.00)	0.027

*Note*: Model 1: unadjusted; Model 2: adjusted for age, education, income, and lifestyle factors (smoking status, alcohol consumption, and physical activity); Model 3: fully adjusted for age, education, income, lifestyle factors, family history of stroke, and predefined vascular risk factors (hypertension, dyslipidemia, diabetes mellitus, CVD, overweight/obesity, and carotid artery stenosis).

Abbreviation: VMS, vasomotor symptoms.

*p* value < 0.05 was considered statistically significant.

### 3.4 Correlations Between Modified KI Scores, HFRS Scores, and NIHSS Scores

In the IS group, the median NIHSS score at admission was 3 (IQR: 0.25, 7). Among IS patients reporting VMS within the past week, the median HFRS score was 10 (IQR: 6.25, 14.75). Correlation analysis showed no significant association between NIHSS and either modified KI or HFRS scores (*p* > 0.05). Partial correlation analysis, adjusting for age, education level, annual income, alcohol consumption, smoking status, physical activity level, CVD, hypertension, family history of stroke, carotid artery stenosis, diabetes mellitus, dyslipidemia, and overweight/obesity, also demonstrated no correlation (*p* > 0.05, Table 4).

**TABLE 4:** Pearson correlation and partial correlation analysis of NIHSS Scores with modified KI and HFRS scores.

	NIHSS (unadjusted)		NIHSS (adjusted)	
	*r*	*p* value	*r*	*p* value
KI	0.101	0.343	0.087	0.666
HFRS	0.171	0.292	0.182	0.364

**Abbreviations**: HFRS, hot flush rating scale; KI, modified Kupperman index; NIHSS, National Institutes of Health Stroke Scale.

*p* value < 0.05 was considered statistically significant.

### 3.5 Predictive Value of VMS for IS Risk

**FIGURE 2:**
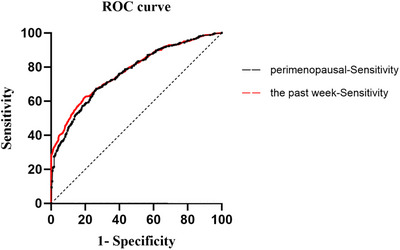
ROC curve of VMS at different periods for IS.

The predictive value of VMS for IS risk was evaluated using ROC curve analysis (Figure 2). As shown in Table 5, the area under the ROC curve (AUC) for both perimenopausal VMS and VMS within the past week was 0.809, indicating a significant predictive value for IS (*p* < 0.001).

**TABLE 5:** Predictive value of VMS at different periods for IS.

	AUC	*p* value	95% CI
VMS during the perimenopausal period	0.809	<0.001	0.77–0.85
VMS in the past week	0.809	<0.001	0.77–0.85

Abbreviation: VMS, vasomotor symptoms.

*p* value < 0.05 was considered statistically significant.

## 4 Discussion

In this multicenter case‐control study, we identified a significant and independent association between VMS and IS, even after adjusting for traditional cardiovascular risk factors, including hypertension, diabetes mellitus, carotid artery stenosis, and physical inactivity. Notably, the strength of the association between hot flashes and IS demonstrated a dose–response relationship, with the strongest effect observed among women experiencing frequent VMS episodes (hot flashes and/or night sweats ≥ 10 times/day) during perimenopause. These findings underscore the clinical relevance of VMS as a potential marker for cerebrovascular risk in midlife women.

### 4.1 Occurrence and Timing of VMS

Previous studies investigating VMS and CVD have typically combined stroke with other cardiovascular outcomes, such as coronary heart disease, myocardial infarction, and heart failure. However, a potential link between VMS and stroke remains discernible. A pooled analysis of twelve prospective cohort studies reported that VMS were associated with a 43% increased risk of stroke after adjustment for age and other confounders (RR: 1.43, 95% CI: 1.07–1.92) (Muka et al. 2016). Similarly, a summary of prospective cohorts from Australia, the United Kingdom, and the United States demonstrated that both premenopausal (HR: 1.38, 95% CI: 1.10–1.75) and postmenopausal (HR: 1.69, 95% CI: 1.32–2.16) VMS were associated with increased CVD risk, including stroke (Zhu et al. 2020). In our cohort, perimenopausal VMS were associated with stroke (OR: 1.93, 95% CI: 1.10–3.38), while postmenopausal VMS were linked to an even higher stroke risk (OR: 2.47, 95% CI: 1.27–4.77).

Age may modify the relationship between VMS and CVD. The Australian Women's Health Study (Dam et al. 2020), which followed 8881 women aged 45–50 years for 20 years, reported no significant association between hot flashes or night sweats and stroke. A subsequent systematic review and meta‐analysis (Armeni et al. 2023) indicated that VMS increased CVD risk primarily among women younger than 60 at baseline, with no significant effect in women aged 60 or older. Although nearly half of the participants in this study were over 60 years of age, a significant association between VMS and stroke was still observed, suggesting that this relationship may persist into the later stages of midlife and beyond.

Studying the relationship between VMS and CVD poses temporal and methodological challenges, as VMS generally occur during midlife, whereas clinical CVD events often manifest later in life (Carson and Thurston 2023). Consequently, many studies rely on retrospective recall of midlife VMS in older women, potentially introducing age and recall bias that may contribute to inconsistent findings.

### 4.2 VMS Frequency and Duration

Evidence from longitudinal cohorts further highlights the importance of VMS frequency and duration. The Study of Women's Health Across the Nation (SWAN) (Thurston et al. 2021) defined frequent VMS as occurring ≥ 6 days/2 weeks and persistent VMS as averaging four episodes. Midlife frequent VMS (HR: 1.51, 95% CI: 1.05–2.17) and persistent VMS (HR: 1.77, 95% CI: 1.33–2.35) were associated with increased risks of later myocardial infarction, stroke, heart failure, and revascularization. Another trajectory‐modeling study (Kim et al. 2024) found that women with a history of migraine and persistent VMS exhibited the highest stroke risk (HR: 3.15, 95% CI: 1.35–7.34). Conversely, some studies have suggested that VMS severity, rather than frequency, may better predict cardiovascular outcomes (Zhu et al. 2020).

Frequency, duration, and severity of VMS are influenced by menopausal status and hormone therapy use. We excluded participants on hormone therapy or with hormone‐dependent tumors. We treated perimenopausal and recent (past week) VMS frequency as categorical variables (<3, 3–9, and ≥10 times/day) and assessed VMS severity using the HFRS. Compared with women without VMS, those reporting ≥3 times/day exhibited higher odds of IS, whereas the bothersomeness of VMS was not significantly associated with stroke severity.

### 4.3 Potential Mechanisms

Several plausible mechanisms may underlie the observed association between VMS and IS. Although hypertension, obesity, diabetes, and dyslipidemia predispose women to both VMS and IS (Eng et al. 2024; Franco et al. 2015; Kagitani et al. 2014; Li et al. 2021), our findings persisted after adjustment for these factors, suggesting additional pathways. The rapid decline and fluctuation of estrogen during the menopausal transition attenuate its vascular protective effects, including vasodilatory, anti‐inflammatory, antioxidant, and metabolic (Raz 2014), thereby increasing the risks of hypertension and atherosclerosis. Subclinical CVD markers, such as white‐matter hyperintensities and increased carotid intima‐media thickness (Thurston et al. 2016; Thurston et al. 2023), may also interact with VMS to further elevate stroke risk.

During hot flash episodes, transient elevations in skin blood flow and fluctuations in blood pressure may promote vascular remodeling and accelerate arterial stiffening. Additionally, VMS may be a manifestation of underlying systemic inflammation or impaired vascular endothelial function (Thurston et al. 2018; Wu et al. 2025), which can facilitate thrombus formation and vascular occlusion, key pathophysiological processes underlying IS. These mechanisms collectively provide a biological basis for the observed association between frequent VMS and elevated stroke risk.

### 4.4 Limitations

First, by excluding women over 70 years old, we minimized recall bias but were unable to assess long‐term stroke risk extending beyond two decades after VMS onset. Second, VMS were self‐reported. Although objective measures exist, such as sternal skin conductance, their accuracy and feasibility remain debated, and they may not outperform subjective reporting (Pachman et al. 2013). Third, the cross‐sectional design with a relatively small sample size limits generalizability and precludes causal inference. Fourth, residual confounding by unmeasured factors, such as migraine history or adverse pregnancy outcomes, cannot be excluded. Future large‐scale longitudinal studies are warranted to confirm these associations and elucidate causality.

## 5 Conclusions

The occurrence and frequency of VMS independently predict ischemic stroke risk in a dose‐dependent manner. Women experiencing frequent or severe VMS may benefit from targeted interventions, as well as vigilant stroke prevention and early screening, to mitigate their elevated cerebrovascular risk.

## Author Contributions

**Xiaoqing Jiang**: conceptualization, methodology, investigation, data curation, writing – original draft. **Qi Qiu**: conceptualization, methodology, data curation, writing – original draft. **Yue Wang**: investigation, formal analysis, visualization. **Yan Xie**: investigation, formal analysis. **Yuanmei Zhao**: investigation, data curation. **Ling Yuan**: investigation, data curation. **Chunyu He**: supervision, writing – review and editing, project administration.

## Funding

This study was funded by the Chengdu Medical College graduate research innovation fund project in 2024(YCX2024‐01‐92).

## Conflicts of Interest

The authors declare conflicts of interest.

## Consent

Before inclusion in the study, all participants signed informed consent under the principles of the Declaration of Helsinki.

## Data Availability

The datasets generated during and/or analyzed during the current study are available from the corresponding author on reasonable request.
